# Mothers in Same-Sex Relationships Describe the Process of Forming a Family as a Stressful Journey in a Heteronormative World: A Swedish Grounded Theory Study

**DOI:** 10.1007/s10995-018-2525-y

**Published:** 2018-05-08

**Authors:** Heléne Appelgren Engström, Elisabet Häggström-Nordin, Catrin Borneskog, Anna-Lena Almqvist

**Affiliations:** 1 School of Health, Care and Social Welfare, Mälardalens University, Västerås, Sweden; 2 Department of Medical and Health Science, Linköpings University, Linköping, Sweden; 3 School of Health, Care and Social Welfare, Mälardalens University, Eskilstuna, Sweden

**Keywords:** Antenatal care, Assisted reproduction technique, Women in same-sex relationships, Parental support

## Abstract

*Objectives* The aim of this study was to gain insight into how women in same-sex relationships experience the process of forming a family through the use of assisted reproduction technique (ART), from planning the pregnancy to parenthood, and their experience of parental support from healthcare professionals. *Methods* The participants were 20 women in a same-sex relationship who had conceived through ART at a Swedish clinic. Semi-structured interviews including open questions about pregnancy, parenthood and support from healthcare professionals were conducted. The interviews were tape-recorded and transcribed verbatim. The data were analysed according to grounded theory. *Results* The core category, *A stressful journey through a heteronormative world*, emerged from the analysis, as did three subcategories: *A journey fraught with difficulties and decisions; The nuclear family as the norm*; and *A need for psychological support*. Same-sex parents expressed a need for more information about how to access ART in Sweden. Both the healthcare organization and treatment were perceived as heteronormative. In particular, these women lacked psychological support during the demanding process of utilizing a sperm donor to conceive. *Conclusions for Practice* Professionals in antenatal care should undergo mandatory cultural competency training to ensure cultural sensitivity and the provision of updated information, tailored brochures and early parental support for families with same-sex parents. All parents need guidance and support from competent, caring personnel throughout the entire process of forming a family.

## Significance

*What is already known on this subject?* Families with two mothers are becoming increasingly common; therefore, healthcare providers are likely to encounter two-mother families in antenatal care. Previous research shows that professionals in antenatal care use heteronormative language and lack knowledge of the unique experience of two women embarking on parenthood.

*What this study adds?* This study offers insight into how women in same-sex relationships experience the process of forming a family through assisted reproduction at Swedish clinics.

## Introduction

Since 2005, women in same-sex relationships in Sweden have had the legal right to assisted fertilization with semen donated at clinics. Such treatment demands basic medical and psychosocial investigation (Socialstyrelsen 2005). According to Swedish legislation regarding gamete donation, both partners become legal parents with joint custody of the child. Then, upon reaching a mature age, the child has the legal right to obtain information about the donor’s identity (Sveriges Riksdag 2006). Good maternity care is a human right (World Health Organization [WHO], n.d.), and the objective of the Swedish healthcare system is to promote good health and deliver care on equal terms (Sveriges Riksdag 2017). The midwife’s role in antenatal care is to monitor maternal and foetal health throughout the pregnancy and provide parental support including helping with preparations for the birth (International Confederation of Midwives [ICM], n.d.; Socialstyrelsen 2014).

U.S. research has reported that women in same-sex relationships can face various issues along the path to parenthood, such as financial security, the need to feel supported and a fear of intolerance (Wall 2011). Couples have described the process of becoming pregnant as stressful and difficult, with many insemination attempts (Goldberg 2006). Similar UK studies have highlighted difficulties in dealing with semen when utilizing a sperm donor to conceive (Nordqvist 2011, 2012). Studies in other Western countries have reported mostly positive experiences with healthcare personnel, although the information and care tended to be heteronormative (Johnson and Nemeth 2014; O’Niell et al. 2013; Wojnar and Katzenmayer 2014).

Although Swedish society is considered to be egalitarian, many people make heteronormative assumptions, and this becomes apparent when same-sex couples meet with healthcare professionals to discuss pregnancy and childbirth. In addition to the language that healthcare professions tend to use, antenatal classes are perceived as gender-stereotypical and heteronormative (Larsson and Dykes 2009; Malmquist and Nelson 2014; Röndahl et al. 2009). Moreover, several researchers have emphasized the importance of the non-birth mother’s recognition as a parent (Dahl and Malterud 2015; Erlandsson et al. 2010; Larsson and Dykes 2009). However, same-sex couples trying to form a family in Sweden have also reported good psychological health and low parental stress (Borneskog et al. 2013, 2014).

Owing to changes in the law concerning access to assisted reproduction technique (ART), healthcare providers are likely to encounter same-sex couples in antenatal care. Studies have shown that antenatal and child healthcare personnel lack competence in providing culturally sensitive care. Thus, the aim of this study was to gain insight into how women in same-sex relationships experience the process of forming a family through assisted reproduction, from planning the pregnancy through to parenthood, and the parental support they receive from healthcare professionals.

## Method

The study employed grounded theory (GT), as this method is aimed at generating explanations of social processes and formulating a preliminary model grounded in empirical data (Corbin and Strauss 2008).

### Setting and Participants

Nurses at a child healthcare clinic distributed a letter describing the study to prospective participants, and the information was posted on a webpage for same-sex families. Interested parties contacted the first author for further information. The inclusion criteria were birth mothers and non-birth mothers in a same-sex relationship, who had had a child through assisted reproduction at a Swedish clinic, with the child being around 1–3 years, and the parents having joint custody and living in mid-Sweden. In line with GT, the sampling procedure was purposeful and theoretical. Purposeful sampling is intended to produce maximum diversity (Corbin and Strauss 2008); therefore, the study targeted participants from both rural and urban areas. Theoretical sampling is concept-driven; thus, data collection continued until saturation was reached (n = 20) and no new data emerged. Eight couples and an additional four birth mothers participated. These 12 birth mothers and 8 non-birth mothers ranged in age from 25 to 42 years (mean = 34 years). Twelve participants had one child and eight had two or more children. Thirteen participants were married and seven were cohabiting. The lengths of their relationships varied from 4.5 to 13 years (mean = 8.5). Fourteen participants had a university degree and six had a high school diploma.

### Procedure and Data Analysis

An interview guide with open-ended questions was constructed according to the themes of *planning for parenthood, pregnancy, childbirth* and *parental support*. The analysis was ongoing; when data generated new questions, they were addressed in the next interview. The longer the interview process was, the more detailed the questions became. The first author conducted each interview in Swedish in a quiet place of the participant’s choosing, such as in her home or at her workplace. The interviews began with the open-ended question, ‘Please tell me how you thought and reasoned about your decision to form a family’. The interviews lasted between 35 and 70 min and were recorded and transcribed verbatim.

**Fig. 1:**
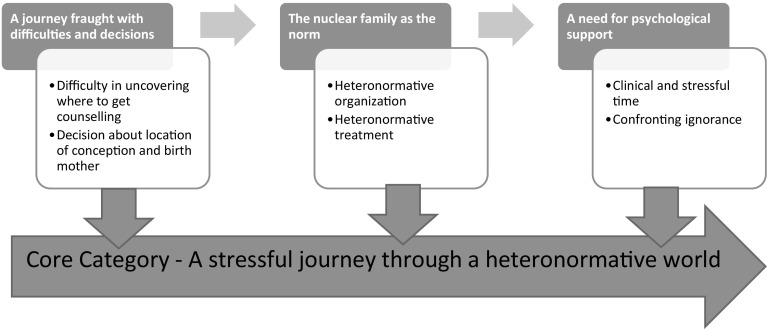
Process of forming a family through assisted reproduction for women in same-sex relationships

A three-step constant comparative analysis was conducted. The first step was *open coding*, where the material was read line by line to identify codes, which were compared and sorted to form categories. Data collection and analysis occurred simultaneously and the process was ongoing (Corbin and Strauss 2008). The second step, *axial coding*, involved coding around each category to determine its properties and the relationships between categories and subcategories. The last step, *selective coding*, was aimed at reaching saturation of the categories and subcategories (see Table 1) and defining and linking categories around the core category (see Fig. 1) (Corbin and Strauss 2008). The analysed material was divided in two parts: (i) The experience of the process of forming a family and (ii) The experience of parenthood. This article describes the former.

**Table 1:** Examples of codes, subcategories and categories that emerged from the results

Open code	Subcategory	Category
Always wanted a child Difficult to obtain information Did not get support	Difficulty in uncovering where to get counselling	A journey fraught with difficulties and decisions
Swedish clinic felt safe Both become legal parents Wanted to be pregnant No desire to give birth	Decision about location of conception and birth mother	

Theoretical memos were written throughout the process and were used to link and verify analytical interpretations of the data.

### Research Ethics

The Swedish Ethical Board in Uppsala approved this study (Dnr.2014/514). The participants were informed orally and in writing about the study and were guaranteed confidentiality. Further, they understood that participation was voluntary and could be withdrawn at any time. All participants gave written informed consent.

## Results

### A Stressful Journey Through a Heteronormative World

Participants described the process of forming a family through ART as *a stressful journey through a heteronormative world*, which formed the core category. The three subcategories were (i) *A journey fraught with difficulties and decisions*, (ii) *The nuclear family as the norm* and (iii) *A need for psychological support*. Participants did not know where to turn to receive information about, or support with, ART. They described healthcare professionals and treatment guidelines as heteronormative, and the transition to parenthood as stressful and lacking in support.

### A Journey Fraught with Difficulties and Decisions

Participants described their desire to have a child and difficulty in knowing where to get counselling. One participant had asked the midwife at a cell sampling, ‘If we want to have children, where do we turn? How do we do it?’ The midwife had no answers: ‘She had no idea, so she referred us to a private gynaecologist.’ Another participant described searching online for information and then phoning a women’s clinic: It was actually a lousy conversation that I had worried about for several weeks, months—I do not remember anymore—before I called. But it was very short, unpleasant. I wanted information. I had tried to google, but they just said, “If you have not been a couple for two years, you may call back…” Although participants had many questions, they had difficulty getting answers from midwives or staff at antenatal clinics. Some participants felt that these professionals had limited knowledge and were unable to help; in fact, they had had to educate the staff. One participant said, ‘The midwife asked me [the questions] I wanted to ask’. Participants were left without information or someone to guide them through the process.

The journey involved making various decisions, like how and where to access ART and which partner would be the birth mother. In most cases, going to a Swedish clinic was an obvious choice. One participant said, ‘Here in Sweden, the non-birth mother will be the parent directly. If we had done it abroad, she would have had to adopt the child, so it was probably the most important issue for us in why we chose a Swedish clinic’. Almost all participants felt safe about ART in Sweden, as the clinic is responsible for the whole treatment process and the child has the right later on to obtain information about the donor. Another significant factor was the low cost, since ART at Swedish clinics is publically funded. Some participants expressed feeling anxious about being accepted as parents before they started the process: ‘That counsellor meeting was what we were most worried about, because we had heard a lot about that. Think if we were not approved…’ Participants described this meeting in different ways: ‘It felt like she did not really know what her task was’ and ‘She asked us what we were doing there’.

Regarding deciding who would be inseminated, most participants described this as a natural and obvious choice: ‘I wanted to do it’. Some had always wanted to be pregnant and give birth, while others did not. One participant said, ‘I have no interest in being pregnant... I think I have a little fear of childbirth’. Another participant said that it did not matter as long as they got a child. Some couples had taken turns. The women mentioned age and career as factors influencing this decision.

### The Nuclear Family as the Norm

The heteronormativity of the healthcare organization and treatment evinced that the nuclear family was considered the norm. Images of nuclear families were in every brochure and on medical forms. One participant said, ‘There were never same-sex families in the information films... It is not that we felt marginalized, but sometimes, they might have thought that it would have been nice if things were a bit different’. Another participant said, ‘When you get that book from antenatal care, with the pregnant mother and father, you are going to be parents, and then mother and father, and the father’s chapter… and you do not want to open it, just throw it in the garbage. And it points, in a way, at how everything is wrong, wrong, wrong, wrong—at least it feels like that’.

Participants had different experiences with healthcare professionals. Some described their meeting with the midwife as very good, while others felt that they and their partner were not treated as a couple and that the midwife was not prepared to meet a family with two mothers. They also characterized parental groups as heteronormative. One non-birth mother reflected, ‘They talked a lot about mothers and fathers, actually... we would do exercises with mothers and fathers, which would divide us’. Non-birth mothers felt singled out and excluded from the group. One participant even described gendered coffee service: ‘Even the cup we received when the girls were born—a “Mum” mug and a “Dad” mug’. They also encountered a sign in the maternity ward reading, ‘Only fathers and siblings are welcome in the department for visits’. Another participant discussed low expectations: ‘When you get something that says “Parents”, instead of “Mom and Dad”, you feel happy.’

Heteronormativity was also evident in that same-sex couples received the same treatment scheme as infertile heterosexual couples; the fact that they had to undergo a tubal flush and preparation of oocyte treatment with hormone stimulation confused some of the women.

I knew, for example, I would get pregnant easily, but I still had to do these steps, flushing the tubes and taking hormones, so as to be able to control ovulation and so on, and I didn’t think it was so great. I would rather have done it without hormones, and then we also got twins. (Mother of twins)

Participants also stressed how difficult hormone therapy had been to undergo; it sometimes led to over-stimulation and unbearable pain. One participant said, ‘I thought it was pretty tough with that treatment, and I was quite affected’.

### A Need for Psychological Support

Almost all participants perceived the process of conceiving as time-consuming, stressful and clinical, and the healthcare professionals as lacking knowledge. They found the process mentally demanding, with great anticipation and fear of miscarriage—and, if a miscarriage occurred, the process would start again.

Yes, it took quite a while for us. We did all four inseminations, so it was quite intense. And finally, we did In Vitro Fertilisation (IVF), so there were many trips back and forth, wondering what will happen. And so, it was a pretty tough time, really. Everything goes very slowly when you are waiting and hoping.

Some participants described the pregnancy as stressful and out of their control, and nobody asked them how they felt during this process: ‘No one asked; there was no room for “How are you in all this?”’ Participants also described the fertilization process as clinical and unromantic. One participant used the term ‘baby factory’ to describe the fertility clinic. Another participant said that the frozen sperm came from ‘The ice hotel’. In addition, ART was physically and mentally painful.

Participants mostly described the healthcare professionals as friendly and positively curious, but sometimes lacking in knowledge. One participant described midwives as follows:

They had very little knowledge of IVF and artificial insemination, and everything that you go through. It was probably the worst. It felt really weird because it was next-door to the fertility unit, so it felt like a different world, actually. You feel a little left out when they do not know what you went through.

Despite being married, participants had to confirm the non-birth mother’s parental status. One participant said, ‘When I went home, they [healthcare professionals] thought that I didn’t need to contact social services because we are married, and therefore [there was] no need to confirm the legal parental status of the non-birth mother, but that was not true’. Another participant said, ‘Then we got some papers at home stating that I was not really a mother’. She thought that healthcare professionals should provide information to avoid this.

## Discussion

The women in this study described different ways of experiencing the process of planning and becoming a parent, but their common experience was of stressful, heteronormative treatment. Previous research supports that the process towards parenthood is perceived as stressful (Goldberg 2006), although one Swedish study reported low parenting stress during assisted reproduction (Borneskog et al. 2014).

The reasons for pursuing assisted reproduction at a Swedish clinic varied, such as economic reasons and legal rights, in line with the findings of Wall (2011) and Rozental and Malmquist (2014). Another important reason was that both partners would become the child’s legal parents (Statens Offentliga Utredningar 2007). Nevertheless, some non-birth mothers described having to sign a parental certificate, even though they were married. The decision about who would be the birth mother was an easy choice that was based on factors such as age and the desire to become pregnant, in line with Heyman et al. (2015) findings.

The participants experienced both the treatment and healthcare organization as heteronormative, echoing previous research (Malmquist and Nelson 2014; O’Niell et al. 2013; Wojnar and Katzenmayer 2014). Seeing images of the nuclear family everywhere, such as in brochures and on forms, does not help women in same-sex relationships in their parental role. As Erlandsson et al. (2010) and Larsson and Dykes (2009) reported, both partners need support in preparing for their parental role and recognition as parents. Yet mothers in same-sex relationships continue to have to defend and justify themselves as parents (Malmquist and Nelson 2014). Healthcare professionals need to be aware of the diversity in family structures and provide culturally sensitive care. Culturally sensitive care includes the concepts of knowledge, consideration, understanding, respect and tailored care (Foronda 2008), which reflect the study participants’ experiences. Participants struggled to obtain information about where to access ART. The provision of adequate information and support is critical. Healthcare workers in Sweden should be able to guide women in same-sex relationships, as they have had the right to assisted fertilization for more than 10 years (Socialstyrelsen 2005).

Caring for others is a way to avoid stereotyping and be culturally sensitive, as it involves taking people’s identities into consideration with the desire to understand and respect their needs (Foronda 2008). Healthcare professionals must listen to women’s needs. Another important aspect of delivering sensitive care is understanding that hormone therapy can cause serious side effects, one of which is getting pregnant with twins (Grainger et al. 2006)—which some participants in this study had experienced.

Participants described their one-on-one encounters with midwives as satisfying, but their treatment in parental groups as unsatisfactory. In fact, they sometimes avoided parental groups, despite their need for support. These groups should support parents during pregnancy and early parenthood, as this is important both for the parents and the unborn baby (ICM, n.d.). Tailored parental groups and materials adapted for same-sex parents are other ways of providing culturally sensitive care (Foronda 2008) on equal terms (Sveriges Riksdag 2017).

The need for psychological support was evident during this stressful and clinical process, as participants had experienced loneliness. Good quality healthcare that provides a sense of security is essential (Sveriges Riksdag 2017). To provide quality care as prescribed by the WHO and local laws (Sveriges Riksdag 2017), healthcare professionals must possess knowledge of different treatments and feel confident in their role. The transition to parenthood is a delicate process that stress and heteronormativity can further complicate.

### Strengths and Limitations of the Study

This study’s findings offer insight into how women in same-sex relationships experience the process of childbirth through assisted reproduction. To increase transferability and credibility (Lincoln and Guba 1985), participants were recruited from both rural and urban areas with a geographical spread. To test the transferability, the preliminary model must be tested in different contexts. The first author did the transcription and open coding of all interviews, and the co-authors read the transcripts and coded the interviews separately to ensure credibility and dependability (Lincoln and Guba 1985). To ensure confirmability, the first author discussed the grouping and categorization of codes with the co-authors, who are experienced in GT and have knowledge of women in same-sex relationships forming families. Finally, since the data were collected and analysed in Swedish, but presented in English, this could represent a limitation.

## Conclusion and Implications

Healthcare professionals need to be aware that patients perceive both the organization and treatment as heteronormative. Furthermore, more information is necessary about how to access assisted reproduction in Sweden. Conceiving through ART is a demanding process, and the study participants lacked psychological support. Professionals in antenatal care require mandatory cultural competency training to ensure the delivery of culturally sensitive care and updated and tailored informational brochures, as well as early parental support that is inclusive of families with same-sex parents. Everyone requires guidance and support from competent and caring personnel throughout the entire process of becoming parents.
